# Is accurate routine cancer prognostication psychologically harmful? 5-year outcomes of life expectancy prognostication in uveal melanoma survivors

**DOI:** 10.1007/s11764-021-01036-4

**Published:** 2021-04-19

**Authors:** Stephen L. Brown, Peter Fisher, Laura Hope-Stone, Bertil Damato, Heinrich Heimann, Rumana Hussain, M. Gemma Cherry

**Affiliations:** 1 Department of Primary Care and Mental Health, University of Liverpool, Liverpool, L69 3GB UK; 2 Liverpool Ocular Oncology Centre, Liverpool University Hospitals NHS Foundation Trust, Liverpool, UK; 3 Nuffield Laboratory of Ophthalmology, Nuffield Department of Clinical Neurosciences, University of Oxford, Oxford, OX3 9DU UK

**Keywords:** Prognostication, Uveal melanoma, Anxiety, Depression, Quality of life, Oncology

## Abstract

**Purpose:**

Prognostication in cancer is growing in importance as increasingly accurate tools are developed. Prognostic accuracy intensifies ethical concerns that a poor prognosis could be psychologically harmful to survivors. Uveal melanoma (UM) prognostication allows survivors to be reliably told that life expectancy is either normal (good prognosis) or severely curtailed because of metastatic disease (poor prognosis). Treatment cannot change life expectancy. To identify whether prognosis is associated with psychological harm, we compared harm in UM survivors with good and poor prognoses and those who declined testing and compared these outcomes to general population norms.

**Methods:**

Non-randomized 5-year study of a consecutive series of 708 UM survivors (51.6% male, mean age 69.03, *SD*=12.12) with observations at 6, 12, 24, 36, 48 and 60 months. We operationalized psychological harm as anxiety and depression symptoms, worry about cancer recurrence (WREC) and poor quality of life (QoL).

**Results:**

Compared to other groups, survivors with poor prognoses showed initially elevated anxiety and depression and consistently elevated worry about local or distant recurrence over 5 years. Good prognoses were not associated with outcomes. Generally, no prognostic groups reported anxiety, depression and WREC or QoL scores that exceeded general population norms.

**Conclusions:**

Using a large sample, we found that harm accruing from a poor prognosis was statistically significant over 5 years, but did not exceed general non-cancer population norms.

**Implications for Cancer Survivors:**

Survivors desire prognostic information. At a population level, we do not believe that our findings show sufficiently strong links between prognostication outcome and psychological harm to deny patients the option of knowing their prognosis. Nonetheless, it is important that patients are informed of potential adverse psychological consequences of a poor prognosis.

## Background

Prognostication is evidence-based prediction and communication of future health outcomes [1]. Cancer prognostication after primary treatment can inform adjuvant approaches, alleviate uncertainty or simply bring survivors relief through an optimistic prognosis [2]. Patients frequently show interest in prognostication [3]. Researchers are striving for greater prognostic accuracy through biomarker identification, gene profiling and cytogenetic analysis [4–8]. In some cancers, trustworthy life-expectancy estimates are available [9]. However, accuracy accentuates the ethical conflicts inherent to prognostication [10]. Reliable knowledge of poor prognosis is feared to be psychologically harmful to survivors, causing ethical conflicts for practitioners in developing and offering it and personal conflicts for patients in accepting it [11].

Huntington’s disease (HD) provides the sole existing paradigm for most work on the ethics of accurate prognostication [11], because testing is deterministic with a poor prognosis being severe and irreversible [11, 12]. A good HD prognosis can relieve worry, but a poor prognosis is associated with profound and enduring psychological distress and deterioration in quality of life (QoL) [12]. However, transferability of the HD paradigm into cancer is uncertain because the course of the illness, younger patient age and reproduction implications differ [11]. Further, HD testing is often used diagnostically whereas cancer prognostic testing is unlikely to be so used. However, uveal melanoma (UM) prognostication is similarly accurate, with consequences severe and irreversible, thus providing a similar paradigm to HD, but which is applicable to cancer survivors [11]. To examine potential psychological harms from poor prognoses in cancer, we examined anxiety, depression, worry about cancer recurrence (WREC) and QoL in UM survivors.

### Uveal melanoma as a paradigm for prognostic outcomes

Uveal melanoma is a treatable ocular cancer with 40–50% 10-year mortality [5], attributable to metastatic disease, which is almost always fatal. Those without metastatic disease have an almost normal life expectancy [5]. Metastatic disease develops almost exclusively in survivors whose tumour cells show deletion of one of the normal two copies of chromosome 3 (monosomy 3 [M3]); disomy 3 (D3) is the normal condition, with both maternal and paternal copies of chromosome 3 [6]. M3 confers a poor prognosis with 10-year disease-specific mortality over 50% (if tumour diameter is >12 mm), whilst the D3 mortality rate is close to 0% [6]. At the time of this study, no treatments prolonged life for M3 survivors. Prognostic testing in UM is controversial, with many practitioners not offering testing due to their concerns about psychological harm [13].

Current studies of psychological harm caused by prognostic testing outcomes show mixed findings. In a sample of 99 survivors, Beran et al. [3] found no indication of greater depressive symptoms or lower QoL associated with a poor prognosis compared to a good prognosis. In 175 survivors with follow-up exceeding 12 months, Lieb et al. [14] found that trends in distress and anxiety and depression symptoms did not differ between survivors with good and poor prognostic outcomes or those who declined testing. However, only 63 survivors accepted testing. Between-group comparisons in this and the Beran et al. study may be underpowered. In a larger sample of 411 survivors, Hope-Stone et al. [15] showed higher depression scores in M3 survivors over 2 years post-diagnosis, particularly in younger and female survivors. M3 survivors’ depression means did not exceed community norms for similar ages. Currently, there are no highly powered studies in the published literature with follow-up greater than 2 years that examine the risks of psychological harm to UM survivors receiving differing chromosome 3 testing outcomes.

### Study Objectives

We assessed whether poor prognosis is associated with long-term psychological harm in an UM survivor population. A secondary interest was the extent to which good prognoses provided protection from harmful outcomes. We operationalized psychologically harmful outcomes as elevated anxiety and depression symptoms, worry about local and metastatic recurrence and lower QoL. We compared survivors from good (D3) and poor prognostic (M3) groups and those who were not tested. Objectives were to (1) test the hypotheses that poor prognosis is associated with psychological harm over the subsequent 5 years compared to other groups and that good prognosis is associated with less harmful outcomes than other groups, controlling demographic and treatment variables; and (2) estimate the magnitude of any harm by comparing outcomes to general population norms.

## Methods

### Participants and Procedure

Data came from a monitoring programme with patient consent for research use, reviewed by the Liverpool Central Ethics Committee (03/06/072/A) consistent with the Declaration of Helsinki. We sampled consecutive adult patients from England or Wales treated for posterior (choroid or ciliary body) UM between April 1^st^ 2008, and December 31^st^ 2014, at the Liverpool Ocular Oncology Centre (LOOC). Treatment was based on a protocol described by Damato and Heimann [13]. Ruthenium plaque radiotherapy or proton beam radiotherapy was first considered. If the tumour was unsuitable for radiotherapy, patients underwent trans-scleral local resection, trans-retinal endoresection or enucleation (eye removal). Prognostic testing was offered and explained to patients during their initial medical consultation. Testing was routinely further explained by nursing staff and supported by printed materials provided both before and after the initial consultation. A psychologist also routinely offered counselling to assist decision-making. Results were explained by LOOC staff or their clinical oncology team. Each prognosis was communicated as a 10-year survival estimate.

After diagnosis, a consecutive series of patients were approached. Patients who gave written consent were posted questionnaires 6, 12, 24, 36, 48 and 60 months after diagnosis with postage-paid return envelopes. Treatments were completed, and test results communicated before the 6-month observation.

### Measures

Demographic and treatment variables were collected from clinical records. The process of prognostic estimation and communication at the time of the study is described by Damato and Heimann [13] and almost wholly determined by chromosome 3 status [6, 9]. For analysis, chromosome 3 testing outcomes were categorized as M3, D3, patient not tested (almost all declined, a small number were not offered testing) and testing failed (tissue specimen insufficient for analysis). Death records for England and Wales were electronically searched by full name and date of birth.

Anxiety and depression symptoms were assessed using the Hospital Anxiety and Depression Scale (HADS) [16]. Each subscale has seven items scored from 0 to 3 (range 0–21) with higher scores signifying greater symptomology. Both subscales predict diagnosed cases with high sensitivity and specificity [17].

Worry about recurrence was assessed using a four-item subscale of the European Organization for Research and Treatment for Cancer Ophthalmic Quality of Life questionnaire [18], validated for UM survivors [19]. Items pertained to local and distant recurrence. Response format was ‘Not at all’, ‘A little’, ‘Quite a bit’ and ‘Very much’, scored 1–4, respectively. Higher scores indicate greater WREC. An item pertaining to loss of the eye was excluded as not relevant to enucleated patients.

QoL was measured using the total score from the 27-item Functional Assessment of Cancer Therapy scale (FACT-G) [20, 21]. Each item is rated on a 5-point scale (0 = not at all; 5 = very much), with total scores ranging from 0 to 108 and higher scores indicating better QoL.

### Analysis

#### Objective 1

We examined whether 5-year trajectories of each harm outcome differed between prognostic groups. Trajectories were estimated using growth-curve modelling (Amos v27) which allows precise modelling of complex time series data [22]. Trajectories are modelled as an intercept, an estimate of the initial score and slope, a model of 5-year changes from the intercept. A positive slope score indicates growth. We used two dummy variables to predict intercept and slope scores. The first dummy variable represented a comparison between poor prognosis and the other groups and the second a comparison between a good prognosis and the other groups.

Demographic, clinical and treatment variables can lead to confounding and thus should be controlled. Covariates were selected from variables that formed univariate predictors of intercept or slope in pilot analyses or previous studies. Younger age, female gender and having been treated by enucleation differed according to prognostic group and were included in the final models as covariates.

The following parameters were initially imposed on the model: observed error variance constrained to equality, mean intercept and slope constrained to zero, intercept and slope constrained to equality and intercept and slope covariance constrained to zero. Parameters were relaxed in the above order until good fitting models (CFI>.95, RMSEA<.05) identified. Linear and quadratic slope models were initially tested with respective parameters 0, 0.5, 1, 2, 3, 4 and 0, 0.5, 1, 2, 4, 8. Slope parameters were then altered to improve model fit until good fit reached. Covariates and predictor variables were then added to these analyses to predict intercept and slope.

#### Objective 2

We compared anxiety, depression and QoL means in all groups to population norms using single-sample *t*-tests. We used Crawford et al.’s [23] (*n*=1,792) UK general population HADS norms of 6.14 for anxiety and 3.68 for depression. We could not find UK HADS norms for older people, instead using Spinhoven et al.’s [24] Netherlands norms for 57–98-year-old individuals (3.90 for anxiety and 4.16 for depression). Community FACT-G norms from Australia [25] (85.42 age range 25–75 *n*=2,719) and Austria [26] (86.5 ages 18–70 *n*=926) were used for comparison. No norms were available for WREC.

#### Missing data strategy

**Fig. 1:**
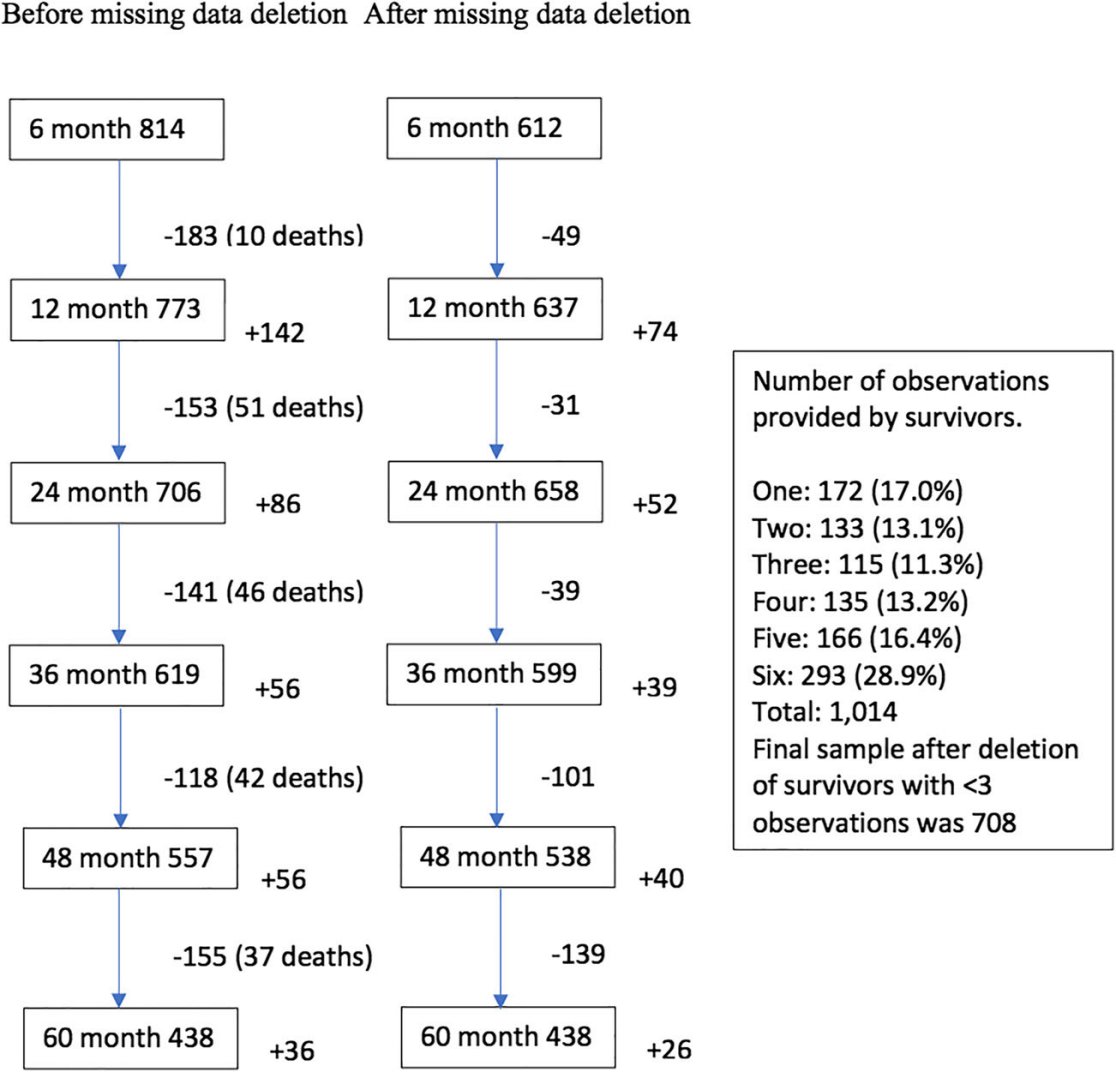
Participant numbers per observation, with numbers absent from (−) and not present at (+) previous observation

Figure 1 shows participants retained, leaving and entering the study at each time point. A total of 1374 patients met inclusion criteria during the study, of whom 1014 survivors provided data at least once. Missing observations were associated with higher anxiety (*r*=−.10, *p*<.01) and depression (*r*=−.15, *p*<.01) but no other study variables. We used a three-step process for data replacement. First, we deleted and did not replace data for 305 cases who contributed less than three of the six time points, including 107 patients (62 (57.94%) were M3) who had died before the third time point. Second, data was estimated for cases that had missed time points but later returned. Data replacement used unbiased full information likelihood estimation (FILA) based on adjacent data points (observed outcome variables) covariates and predictors [27]. Third, missing data was estimated for true attrition (defined as participants who dropped out and did not return for two or more consecutive time points) through a pattern-matching approach using dummy variables coded for dropout occasion as covariates [28]. Replacement was 112 cases at 6 months, 83 at 12 months, 63 at 24 months, 120 at 36 months, 180 at 48 months and 290 at 60 months.

## Results

The final sample, characterized in Table 1, was 708.

**Table 1:** Sample characteristics

Characteristic	Total	D3 179 (25.3%)	M3 192 (27.1%)	No Test 296 (48.1%)	Fail 41 (5.8%)
Age	69.03 (12.12)	66.35 (12.35)	67.85 (12.04)	71.26 (11.64)	70.92 (12.08)
Gender					
Male	363 (51.6%)	95 (26.2%)	109 (30.0%)	139 (38.3%)	20 (5.5%)
Female	340 (48.4%)	84 (24.7%)	83 (24.4%)	153 (45.0%)	20 (5.9%)
Marital status					
With partner	528 (75.1%)	144 (27.3%)	145 (27.5%)	216 (40.9%)	23 (4.4%)
Separated	44 (6.3%)	9 (20.5%)	16 (36.5%)	13 (29.5%)	6 (13.6%)
Widowed	86 (12.2%)	15 (17.4%)	20 (23.3%)	43 (50.0%)	8 (9.5%)
Single	44 (6.3%)	11 (25.0%)	11 (25.0%)	19 (43.2%)	3 (6.8%)
Employment status					
Employed	258 (36.8%)	76 (24.5%)	71 (27.5%)	96 (37.2%)	15 (5.8%)
Homemaker	24 (3.4%)	8 (33.5%)	4 (16.7%)	11 (45.8%)	1 (4.2%)
Retired	348 (49.6%)	70 (20.1%)	95 (27.3%)	164 (47.1%)	19 (5.5%)
Unemployed	8 (1.1%)	3 (37.5%)	2 (25.0%)	2 (25.0%)	1 (12.5%)
Sick leave	33 (4.7%)	11 (33.3%)	9 (27.3%)	11 (33.3%)	2 (6.1%)
Student	8 (1.1%)	3 (37.5%)	0 (0%)	3 (37.5%)	2 (25.0%)
Primary treatment					
Plaque radio	327 (46.2%)	70 (21.4%)	52 (15.9%)	177 (54.1%)	28 (8.6%)
PBR	167 (23.6%)	45 (26.9%)	30 (18.0%)	85 (49.7%)	9 (5.4%)
Enucleation	155 (21.9%)	48 (31.0%)	94 (60.6%)	12 (7.9%)	1 (0.6%)
Resection	34 (4.8%)	13 (39.2%)	16 (47.1%)	4 (11.8%)	1 (2.9%)
Other	25 (3.5%)	3 (12.0%)	0 (0.0%)	20 (80.0%)	2 (8.0%)

### Results overview

Poor prognosis (M3 group membership) was associated with higher intercept scores for anxiety, depression and WREC, indicating that poor prognoses may cause initially higher scores on these variables. Anxiety and depression showed negative associations with slope scores, suggesting that initial gaps closed. Thus, poor prognosis was associated with initially higher anxiety and depression that reduced over time and associated with enduring elevations on WREC. Poor prognosis did not predict QoL. Good prognosis (D3 group membership) did not predict any outcome compared to not tested survivors. Nonetheless, survivors with a poor prognosis did not show markedly higher scores than population norms for any outcome. Thus, whilst survivors with a poor prognosis did experience greater anxiety, depression and WREC, outcomes were generally not worse than those experienced by the general population.

### Anxiety

#### Objective 1

Observed means are presented in Table 2. The full sample trajectory showed a non-significant linear slope (parameters 0, 0.5, 1, 2, 3, 4) indicating that anxiety did not change over time. Table 3 shows good fit for the predictive model (Appendix Fig. 2). A poor prognosis (M3 group) was associated with a significantly higher intercept showing initially higher anxiety in this group. M3 was also associated with a negative slope, showing that this gap significantly reduced over the subsequent 4.5 years. This can be clearly seen on the means displayed in Table 2. D3 group membership was not associated with either intercept or slope, showing no differential anxiety to the rest of the sample or the not tested group.

**Table 2:** Temporal trends for means and *SE*s in outcome variables for the full sample and by prognostic group

	6 mth	12 mth	24 mth	36 mth	48 mth	60 mth	Intercept	Slope	CMIN	RMSEA	CFI
Anxiety											
Full sample	5.36 (.17)	5.16 (.17)	5.15 (.16)	5.17 (.17)	4.98 (.18)	5.00 (.20)	5.29 (.158)	−.04 (.03)	33.91 (16)	.040	.994
D3	5.04 (.32)	4.75 (.31)	4.85 (.32)	4.73 (.34)	4.74 (.34)	4.63 (.35)	5.01 (.28)	−.07 (.06)			
M3	6.15 (.35)	5.77 (.35)	5.35 (.33)	5.53 (.39)	5.12 (.39)	5.16 (.40)	5.78 (.32)	−1.76 (.07)	63.24 (48)	.022	.994
No test	5.02 (.26)	4.94 (.25)	5.11 (.23)	5.22 (.26)	5.11 (.28)	5.32 (.31)	5.07 (.22)	.04 (.04)			
Fail	4.87 (.65)	5.46 (.69)	5.71 (.72)	5.35 (.63)	4.70 (.60)	4.42 (.63)					
Depression											
Full sample	3.16 (.13)	3.25 (.14)	3.23 (.14)	3.41 (.15)	3.23 (.15)	3.28 (.18)	3.23 (.124)	.08 (.03)*	46.58 (16)	.052	.989
D3	2.63 (.22)	2.81 (.25)	2.88 (.25)	2.94 (.27)	2.98 (.27)	3.00 (.32)	2.80 (.20)	.10 (.05)			
M3	3.74 (.26)	3.79 (.28)	3.56 (.28)	3.74 (.33)	3.21 (.28)	3.08 (.36)	3.67 (.247)	.02 (.06)	80.13 (48)	.032	.988
No test	2.95 (.21)	3.08 (.21)	3.15 (.21)	3.52 (.24)	3.37 (.22)	3.57 (.30)	3.09 (.19)	.143 (.04)*			
Fail	3.63 (.63)	3.68 (.70)	3.62 (.57)	3.39 (.60)	3.51 (.59)	3.32 (.61)					
WREC											
Full sample	2.53 (.05)	2.35 (.05)	2.22 (.05)	2.15 (.04)	2.12 (.05)	2.08 (.05)	2.37 (.01)	−.08 (.02)*	56.29 (16)	.939	.060
D3	2.50 (.12)	2.26 (.10)	2.16 (.08)	2.11 (.09)	2.09 (.10)	2.08 (.10)	2.33 (.08)	−.08 (.03)*			
M3	2.73 (.08)	2.49 (.08)	2.37 (.07)	2.36 (.09)	2.23 (.10)	2.39 (.11)	2.54 (.06)	−.07 (.03)*	101.03 (48)	.916	.041
No test	2.37 (.06)	2.31 (.07)	2.18 (.06)	2.09 (.06)	2.09 (.07)	2.02 (.07)	2.30 (.05)	−.07 (.02)*			
Failed test	2.26 (.13)	2.28 (.14)	2.03 (.12)	2.00 (.11)	1.95 (.12)	1.84 (.10)					
QoL											
Full sample	88.76 (.58)	88.99 (.60)	89.30 (.59)	89.17 (.63)	90.37 (.63)	90.55 (0.72)	88.85 (.53)	−.02 (.01)	46.81 (16)	.052	.989
D3	89.50 (1.09)	91.38 (1.07)	91.07 (1.02)	90.83 (1.18)	91.55 (1.28)	91.09 (1.26)	90.48 (.95)	.19 (.19)			
M3	87.36 (1.09)	87.24 (1.19)	88.24 (1.22)	88.19 (1.37)	91.10 (1.15)	91.09 (1.51)	87.58 (.99)	0.13 (.28)	89.65 (16)	.036	.985
No test	89.12 (1.94)	88.89 (0.92)	88.89 (0.95)	88.58 (0.95)	89.01 (1.00)	89.06 (1.20)	88.83 (.82)	−.29 (.18)			
Failed test	89.97 (2.42)	87.62 (2.85)	89.45 (2.25)	89.86 (2.02)	91.37 (2.28)	90.96 (2.40)					

Intercepts and slopes were not calculated for failed tests due to small sample size. *D3* disomy 3, *M3* monosomy 3, *WREC* worry about cancer recurrence, *QoL* quality of life

**Table 3:** Model fit and standardized estimates for M3 as a predictor of intercept and slope of anxiety and depression symptoms, WREC and QoL

	Anxiety		Depression		WREC		QoL	
Model Fit	CMIN=74.97 (37) CFI=.988 RMSEA=.038		CMIN=64.40 (37) CFI=.991 RMSEA=.032		CMIN=86.76 (37) CFI=.949 RMSEA=.044		CMIN=61.99 (37) CFI=.992 RMSEA=.031	
Outcome	Intercept	Slope	Intercept	Slope	Intercept	Slope	Intercept	Slope
Age	−.25*	.18*	.02	.23*	−.20*	.77	.05	−.22*
Sex	.24*	−.02	.09*	−.09	.14*	−.52	.05	−.12*
Enucleation	−.05	.13	.00	.16*	−.02	.32	.05	−.31*
M3	.10*	−.19*	.09	−.17*	.16*	.03	−.06	.22*
D3	−.04	−.08	−.05	−.03	.03	−.31	.05	−.14

*M3* monosomy 3, *D3* disomy 3, *WREC* worry about cancer recurrence, *QoL* quality of life; *p<.05

#### Objective 2

Anxiety was broadly similar to normative values, although all groups were higher than Spinhoven et al.’s^23^ Dutch mean of 3.9 for the 57–99 age groups. The 6–36-month means in the M3 group did not differ to the UK population mean of 6.15, whilst the remainder of the M3 group and all observations of the other groups were lower than the UK population mean (Table 2).

### Depression

#### Objective 1

Observed means are presented in Table 2. The full sample trajectory showed a non-significant slope (parameters 0, 0.5, 1, 2, 3, 4), again indicating invariance of scores over time (Table 3). Table 3 shows good fit for the predictive model (Appendix Fig. 3). M3 group membership was non-significantly (*p*=.088) associated with a higher intercept. When D3 group membership was removed from the model, this parameter achieved significance (*β*=.11, *p*=.021). M3 was also associated with a negative slope, showing that this gap significantly reduced over the subsequent 4.5 years. D3 group membership was not associated with either intercept or slope, showing no differential depression to the rest of the sample or the not tested group.

#### Objective 2

Depression was generally similar to or lower than normative values. The 6–36-month M3 means did not differ from the general UK population mean of 3.68 or the Dutch 57–99 mean of 4.16, but the 48- and 60-month means were lower than both. All means for other groups were lower than the UK or Dutch means.

### WREC

#### Objective 1

Observed means are presented in Table 2. The full sample trajectory also showed a significant negative slope (parameters 0, 0.5, 1, 2, 3, 4), suggesting that WREC declined over 5 years (Table 3). Table 3 shows reasonable fit for the predictive model (Appendix Fig. 4). M3 group membership predicted higher intercept but not slope estimates (Table 3). This shows that patients with poor prognoses reported consistently higher WREC over the subsequent 5 years and that this gap between M3 and other survivors did not close (Table 2). D3 group membership predicted neither intercept nor slope. D3 group membership was not associated with either intercept or slope, showing no differential depression to the rest of the sample or the not tested group.

### QoL

#### Objective 1

Means are presented in Table 2. The full sample trajectory also showed a non-significant slope (parameters 0, 0.5, 1, 2, 3, 4), again indicating invariance of scores over time (Table 3). The predictive model showed good fit (Table 3). Neither M3 nor D3 group membership predicted the intercept. M3 was associated with a positive slope, meaning that QoL improved in this group relative to the remainder of the sample. Table 2 indicates that this improvement occurred from an initial, but non-significant deficit in the M3 group to approximate parity. Overall, there is little evidence of association between prognostic group and QoL at any point.

#### Objective 2

All group showed higher QoL scores than normative values at each observation.

## Conclusions

We examined 5-year trajectories of anxiety and depression symptoms, WREC and QoL scores across UM prognostic groups. Survivors with a poor prognosis (M3 group) showed higher initial anxiety, depression and WREC over 5 years, but the gaps between M3 and other participants in anxiety and depression reduced over the subsequent 4.5 years. At no point did outcome variables in any group exceed population norms. A good prognosis (D3 group) did not influence trajectories; thus, we found no evidence that a good prognosis confers protection from harm.

Previous UM studies [3, 14, 15] have generally not detected greater psychological harm associated with poor prognosis, although Hope-Stone et al. [15] found higher depression scores. Our study was more highly powered, and it is likely that we detected a small effect that had been missed in previous smaller samples. An obvious explanation for this effect is that psychological harm accrues due to a reduced life expectancy, as has been observed in advanced cancer [23, 29, 30]. Other factors may also contribute to our findings. For example, independent of life expectancy, a poor prognosis also brings the probability of future metastatic disease that causes future physical and psychological morbidity and requires physically demanding treatments [31].

Nonetheless, psychological harm associated with a poor prognosis was generally low. We found M3-related elevations of slightly more than a single point on the anxiety and depression and WREC scales and little evidence that survivors’ outcomes differed from community norms. These findings differ substantially from the HD research discussed earlier, where poor prognosis is associated with significant psychological morbidity and good prognosis with relief [12]. These differences suggest that the HD paradigm may be of limited use in cancer prognostication.

Understanding how our findings differed from the HD research requires an understanding of why differences between prognostic groups were small and why no group showed appreciably greater psychological harm than population norms. First, the impact of a good or a poor prognosis may be mitigated if other survivor concerns compete with life expectancy. Most cancer survivors cite pressing issues that are not directly related to life expectancy; such as pain, functional limitation, social connectedness and personal, family and social disruption [32, 33]. This is also true of UM survivors^32^. In our older sample (HD testing patients are typically younger (aged 20–50)), these concerns may reduce the relative importance of life expectancy. Alternatively, survivors may misinterpret their prognosis. Perception of threat is subjective and influenced by personal motivations [34, 35]. One study shows that UM survivors treat prognosis as uncertain and that uncertainty enables them to build hope after a poor prognosis but also to mistrust a good prognosis [31].

A number of cancer survivor studies show similar anxiety, depression and QoL profiles to population norms [36, 37]. The life evaluation literature suggests that a diagnosis can inspire survivors to find benefits, through a re-evaluation of life priorities [38], which can offset the effects of cancer-related stressors. Low harm might also be explained by the specific characteristics of UM, which generally does not cause debilitating symptoms or warrant extensive surgery or chemotherapy treatments.

### Study limitations

Findings could be confounded by external factors. We did not collect pre-treatment baselines, so we do not know whether or not group outcome differences existed before testing. Of the 1374 eligible survivors approached, 706 participants provided sufficient data for analysis (51.38%), and they showed slightly lower anxiety and depression scores than participants omitted. We do not know whether group differences would be enhanced or diminished if all survivors had provided sufficient data to be retained. Finally, UM prognostication is largely deterministic. Our findings should be tested in other cancers, particularly as prognostic advances will probably yield more complex and probabilistic, and possibly less certain, estimates than UM.

### Clinical implications

Contemporary medical practice is rooted in the principle that patients should contribute to decisions about their care [39]. Many want accurate UM prognostication [3]. At a population level, our findings show that prognostication is unlikely to be sufficiently harmful to withhold it or that the development of prognostic tools should be inhibited by concerns about psychological harm.

Nonetheless, routine UM prognostication is not without moral hazard. Patients’ decisions to accept testing have potentially negative consequences for them because poor prognosis is associated with increased anxiety, depression and WREC. Informed consent requires that patients be advised of these risks. Patients may trust that a good prognosis will reduce future negative emotion or worry [33]. Our findings suggest that a good prognosis does not deliver better outcomes than not having the test. Patients will also need to be warned of this.

The large sample and long-term follow-up period in this study provides the most reliable evidence, yet that cancer prognostication can cause some harm for patients who receive poor prognoses relative to other prognostic groups. However, even a poor prognosis is unlikely to be greatly harmful at a population level. With the caveat that our findings may differ for other cancers, we argue that well-informed patients should have the right to receive reliable prognostic estimates, particularly where these can be linked to effective treatments.

## Figures and Tables

**Fig. 2:**
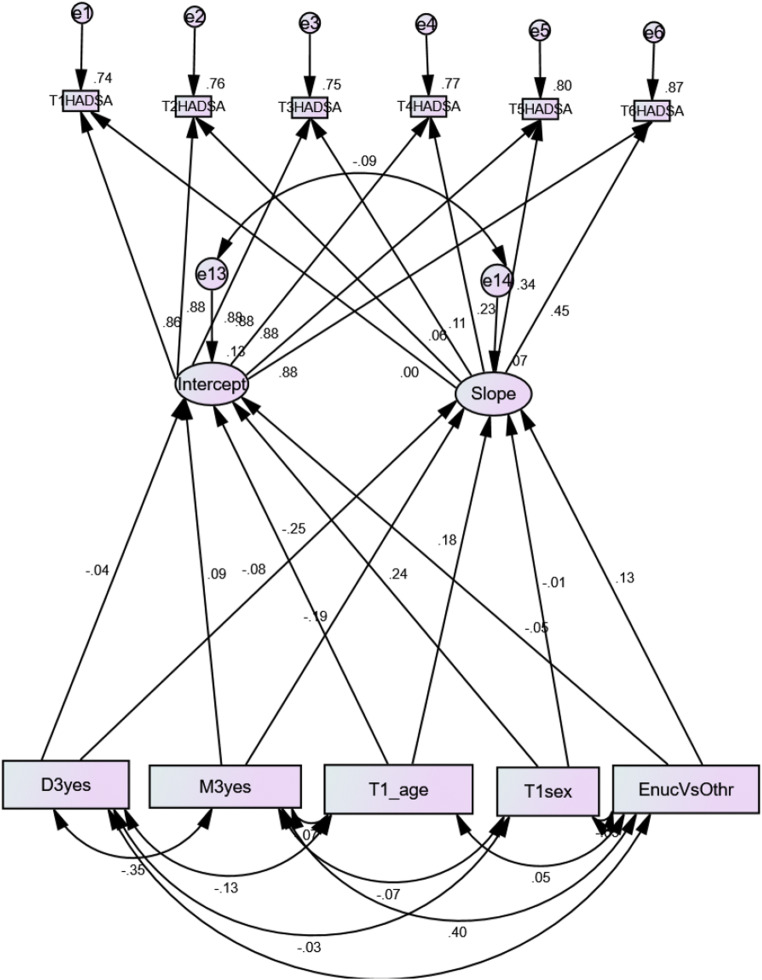
SEM predictive model for anxiety. HADSA=HADS Anxiety; EnucVsOther=Dummy coding for enucleation versus other treatments (Enuc=1, Other=0)

**Fig. 3:**
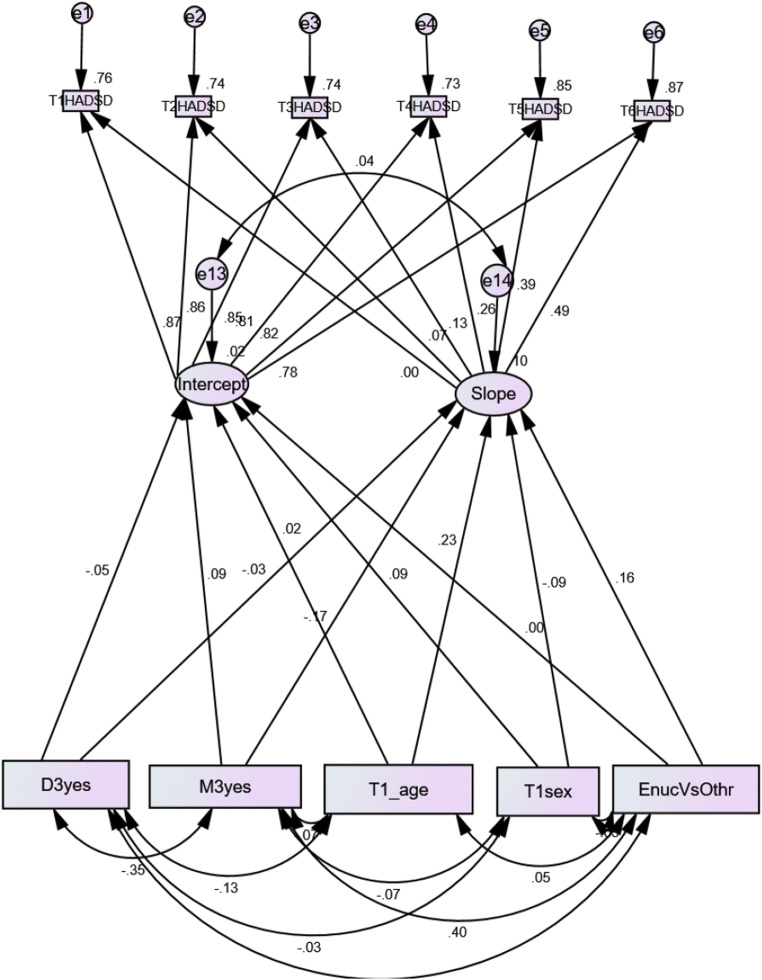
SEM predictive model for depression. HADSD=HADS Depression; EnucVsOther=Dummy coding for enucleation versus other treatments (Enuc=1, Other=0)

**Fig. 4:**
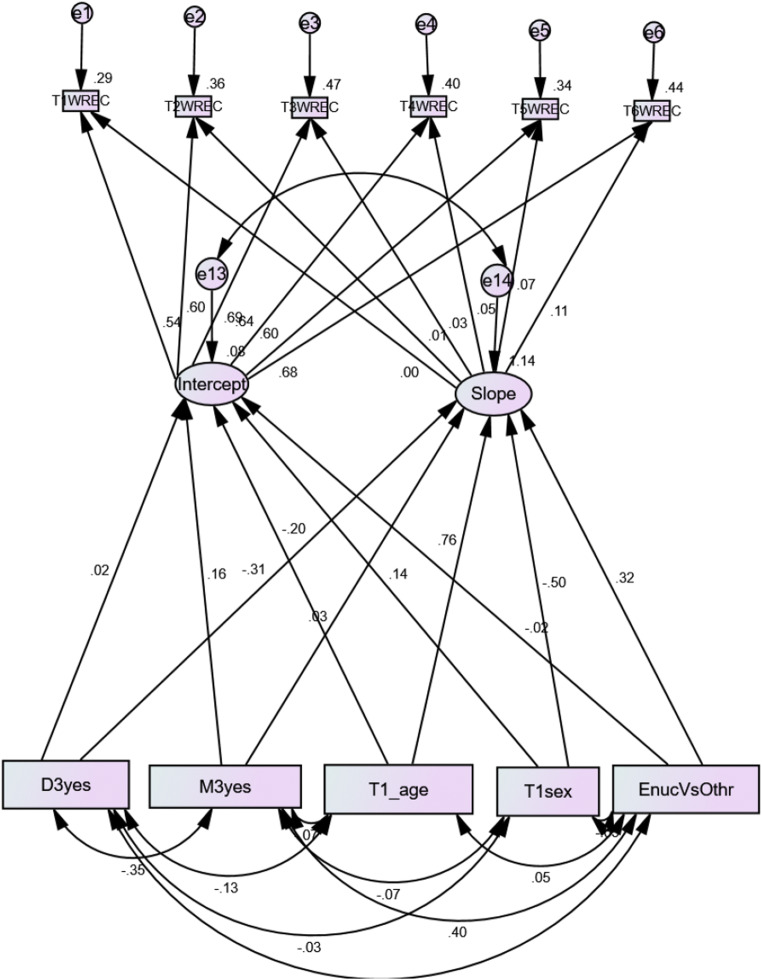
SEM Predictive Model for WREC. WREC=Worry About Cancer Recurrence; EnucVsOther=Dummy coding for enucleation versus other treatments (Enuc=1, Other=0)

**Fig. 5:**
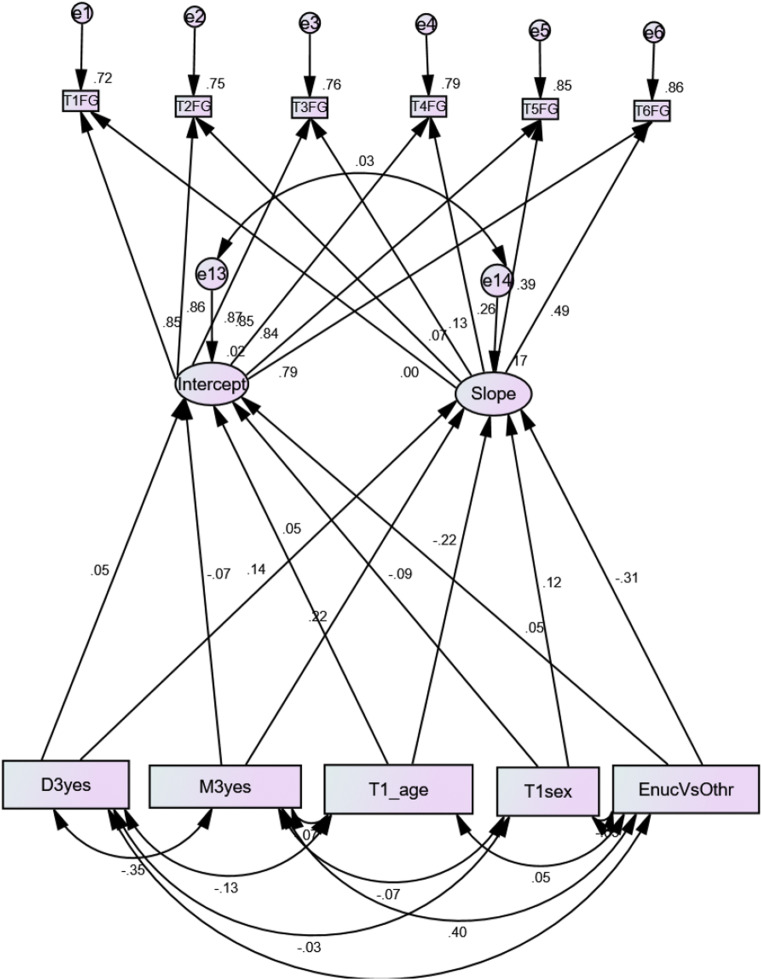
SEM Predictive Model for QoL. FG=FACT G; EnucVsOther=Dummy coding for enucleation versus other treatments (Enuc=1, Other=0)

## Data Availability

Data are available on request from the first author. Ethical clearance may be required before data can be provided.

## References

[1] Kwok S, Salvo N, Pang J, Chow E. Prognostic assessment of the cancer patient

[2] Fahy BN (2019). Prognostication in Oncology. J Surg Oncol.

[3] Beran TM, Mc Cannel TA, Stanton AL, Straatsma BR, Burgess BL (2009). Reactions to and desire for prognostic testing in choroidal melanoma patients. J Genet Couns.

[4] Grinberg M, Djureinovic D, Brunnström HRR, Mattson JSM, Edlund K, Hengstler JG, La Fleur L, Ekman S, Koyi H, Branden E, Ståhle E, Jirström K, Tracy DK, Pontén F, Botling J, RahnenführerJ MP (2017). Reaching the limits of prognostication in non-small cell lung cancer: an optimized biomarker panel fails to outperform clinical parameters. Mod Pathol.

[5] Lønning PE, Knappskog S, Staalesen V, Christanthar R, Lillehaug JR (2007). Breast cancer prognostication and prediction in the postgenomic era. Ann Oncol.

[6] Damato BE, Dopierala JA, Coupland SE (2010). Genotypic profiling of 452 choroidal melanomas with multiplex ligation-dependent probe amplification. Clin Cancer Res.

[7] Yoon EC, Schwattz C, Brogi E, Ventura K, Wen H, Darvishian F (2019). Impact of biomarkers and genetic profiling on breast cancer prognostication: a comparative analysis of the 8^th^ edition of breast cancer staging system. Breast J.

[8] Zhao X, Lingjaerde OC, Børrensen-Dale A-L. Breast cancer prognostication and prediction in the post-genomic era. In: Tyshenko MG, editor. The continuum of health risk assessments: InTech. Available from: http://www.intechopen.com/books/thecontinuum-of-health-risk-assessments/breast-cancer-prognostication-and-risk-prediction-in-the-post-genomicera ISBN: 980-953-307-582-7.

[9] DeParis SW, Taktak A, Eleuteri A, Enanoria W, Heimann H, Coupland SE, Damato BD (2016). External validation of the Liverpool Uveal Melanoma Prognosticator Online. Invest Ophthalmol Vis Sci.

[10] Christakis N (1999). Prognostication and bioethics. Daedalus.

[11] Coustasse A, Pekar A, Sikula A, Lurie S (2009). Ethical considerations of presymptomatic testing for Huntington’s disease. J Hosp Market Pub Relat.

[12] Soldan J, Street E, Gray J, Binedell J, Harper P (2000). Psychological model for presymptomatic test interviews: lessons learned from Huntington’s disease. J Genet Couns.

[13] Damato B, Heimann H (2013). Personalized treatment of uveal melanoma. Eye..

[14] Lieb M, Tagay S, Breidenstein A, Hepp T, Le Guin CHD, Scheel J, Lohmann DR, Bornfeld N, Teufel M, Erim Y (2020). Psychosocial impact of prognostic genetic testing in uveal melanoma patients: a controlled prospective observational study. BMC Psychol.

[15] Hope-Stone L, Brown SL, Heimann H, Damato B, Salmon P (2016). Two-year patient-reported outcomes following treatment of uveal melanoma. Eye.

[16] Zigmond AS, Snaith RP (1983). The Hospital Anxiety and Depression Scale. Acta Psychiatr Scand.

[17] Vodermaier A, Millman R (2011). Accuracy of the Hospital Anxiety and Depression Scale as a screening tool in cancer patients: a systematic review and meta-analysis. Support Care Cancer.

[18] Brandberg Y, Damato B, Kivela T, Kock E, Seregard S (2004). The EORTC Opthalmic Oncology Quality of Life questionnaire Module (EORTC QLQ-OPT30) Development and pre-testing. Eye..

[19] Chmielowska K, Tomaszewski KA, Pogrzebielski A, Brandberg Y, Romanowska-Dixon B (2013). Translation and validation of the Polish version of the EORTC QLQ-OPT30 module for the assessment of health-related quality of life in patients with uveal melanoma. Eur J of Cancer Care.

[20] Cella DF, Tulsky DS, Gray G, Sarafian B, Linn E, Bonomi A, Silberman M, Yellen SB, Winicour P, Brannon J (1993). The Functional Assessment of Cancer Therapy scale: development and validation of the general measure. J Clin Oncol.

[21] Webster K, Yost K (2003). The Functional Assessment of Chronic Illness Therapy (FACIT) measurement system: properties, applications and interpretations. Health Qual Life Outcomes.

[22] Berlin KS, Parra GR, Williams NA (2014). An introduction to latent variable mixture modeling (part 2): longitudinal latent class growth analysis and growth mixture modeling. J Pediatr Psychol.

[23] Crawford JR, Henry JD, Crombie C, Taylor EP (2001). Normative data for the HADS from a large non-clinical sample. Brit J Clin Psychol.

[24] Spinhoven P, Ormel J, Sloekers PP, Kempen GI, Speckens AE, Van Hemert AM (1997). A validation study of the Hospital Anxiety and Depression Scale (HADS) in different groups of Dutch subjects. Psychol Med.

[25] Janda M, DiSipio T, Hurst C, Cella DF, Newman B (2009). The Queensland Cancer Risk Study: general population norms for the Functional Assessment of Cancer Therapy-General (FACT-G). Psychooncol.

[26] Holzner B, Kemmler G, Cella DF, De Paoli C, Meraner V, Kopp M (2004). Normative data for functional assessment of cancer therapy--general scale and its use for the interpretation of quality of life scores in cancer survivors. Acta Oncol.

[27] Nakai M, Ke W (2011). Review of the methods for handling missing data in longitudinal data analysis. Int J Math Anal.

[28] Roy J (2003). Modeling longitudinal data with nonignorable dropouts using a latent dropout class model. Biometrics.

[29] El-Jawahri A, Traeger L, Park ER (2014). Associations among prognostic understanding, quality of life, and mood in patients with advanced cancer. Cancer.

[30] Tang ST, Chang WC, Chen JS (2016). Associations of prognostic awareness/acceptance with psychological distress, existential suffering, and quality of life in terminally ill cancer patients’ last year of life. Psycho-oncol.

[31] Barracliffe L, Yang Y, Cameron J, Bedi C, Humphris G (2018). Does emotional talk vary with fears of cancer recurrence trajectory? A content analysis of interactions between women with breast cancer and their therapeutic radiographers. J Psychosom Med.

[32] Brown SL, Hope-Stone L, Heimann H, Damato B, Salmon P (2018). Effects of post-treatment symptoms, functional problems and worries about recurrent disease on long term anxiety and depression in Uveal Melanoma survivors. Psycho-Oncol.

[33] Hope-Stone L, Brown SL, Heimann H, Damato B, Salmon P (2015). How do patients with uveal melanoma experience and manage uncertainty? A qualitative study. Psycho-Oncol.

[34] Cameron LD, Sherman KA, Marteau TM, Brown PM (2009). Impact of genetic risk information and type of disease on perceived risk, anticipated affect and expected consequences of genetic tests. Health Psychol.

[35] van t’Riet J, Ruiter RA (2013). Defensive reactions to health-promoting information: an overview and implications for future research. Health Psychol Rev.

[36] Osbourne R, Elsworth GR, Sprangers MAG, Ort FJ, Hopper JL (2004). The value of the hospital anxiety and depression scale (HADS) for comparing women with early onset breast cancer with population-based reference women. Qual Life Res.

[37] Herce Lopez J, Rollon Mayordomo A, Lozano Rosado R, Salazar Fernandez CI, Gallana S (2009). Quality of life in long-term oral cancer survivors: a comparison with Spanish general population norms. J Oral Maxillofac Surg.

[38] Helgeson VS, Reynolds KA, Tomich PL (2006). A meta-analytic review of benefit finding and growth. J Consult Clin Psychol.

[39] Barry MJ, Edgman-Levitan S (2012). Shared decision making–pinnacle of patient-centered care. N Engl J Med.

